# Evidence-based periodontal therapy: An overview

**DOI:** 10.4103/0972-124X.44097

**Published:** 2008

**Authors:** R. Vijayalakshmi, V. Anitha, T. Ramakrishnan, Uma Sudhakar

**Affiliations:** 1 *Lecturer, Department of Periodontics, Meenakshi Ammal Dental College and Hospital, Chennai - 600 095, India*; 2 *Reader, Department of Periodontics, Meenakshi Ammal Dental College and Hospital, Chennai - 600 095, India*; 3 *Professor, Department of Periodontics, Meenakshi Ammal Dental College and Hospital, Chennai - 600 095, India*; 4 *Associate Professor, Department of Periodontics, Meenakshi Ammal Dental College and Hospital, Chennai - 600 095, India*

**Keywords:** Evidence, periodontal therapy, study designs

## Abstract

Dentists need to make clinical decisions based on limited scientific evidence. In clinical practice, a clinician must weigh a myriad of evidences every day. The goal of evidence-based dentistry is to help practitioners provide their patients with optimal care. This is achieved by integrating sound research evidence with personal clinical expertise and patient values to determine the best course of treatment. Periodontology has a rich background of research and scholarship. Therefore, efficient use of this wealth of research data needs to be a part of periodontal practice. Evidence-based periodontology aims to facilitate such an approach and it offers a bridge from science to clinical practice. The clinician must integrate the evidence with patient preference, scientific knowledge, and personal experience. Most important, it allows us to care for our patients. Therefore, evidence-based periodontology is a tool to support decision-making and integrating the best evidence available with clinical practice.

## INTRODUCTION

Periodontics is a rapidly changing field with advances in the ability to diagnose, prevent disease and slow its progression, and regenerate lost periodontium. The recent focus is on clinical decision-making and it is our duty to offer the best possible care for patients in an evidence-based manner. Evidence-based approach (EBA) offers a bridge from science to clinical practice.

### What is evidence-based practice?

According to Sacketts, “Evidence-based practice involves integrating individual clinical practice with the best available external clinical evidence from systematic research.”

According to Muir Gray, “An approach to decision-making in which the clinician uses the best available evidence, in consultation with the patient, to decide upon the option which suits the patient best”.[1]

### What is evidence?

Evidence is based on the existence of at least one well-conducted randomized control trial (RCT).

### Need for evidence

The classic example for the need for evidence is William Hunter's focal infection theory which was originally proposed in 1900, but was later discarded in 1940s due to lack of proper evidence.[2] Again the theory was accepted in 1989, due to studies which proved the same with proper evidence.

### Evidence-based decision making

Current efforts focus on producing summaries of studies, and appraising and incorporating the quality of the research. These rigorous analyses are called systematic reviews. If multiple similar studies have been performed, a statistical technique called a meta-analysis is used to combine the results. The clinician can then incorporate the findings of these more powerful tests into decision making.

### Advantages of evidence-based approach compared with other assessment methods

The EBA is:[3]

- Objective.
- Scientifically sound.
- Patient-focused.
- Incorporates clinical experience.
- Stresses good judgement.
- Is thorough and comprehensive.
- Uses transparent methodology.

### What evidence-based practice is not?

**Figure 1:**
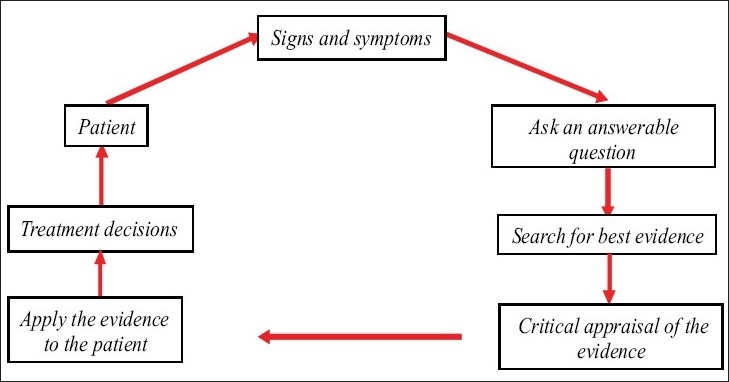
Model of evidence-based approach in clinical practice[5]

It is not simply systematic reviews of RCT, although this is an important aspect. It is an approach to patient care and nothing more. It cannot provide answers if research data do not exist and it cannot substitute for highly developed clinical skills [Figure 1].[4]

### Terminologies used in evidence-based approach[5]

**Systematic review:** Review of a clearly formulated question that attempts to minimize bias using systematic and explicit methods to identify, select, critically appraise and summarize relevant research.

**Interpretation:** It is the process by which qualitative methods seek to identify subjective meaning of a phenomenon.

**Process:** Qualitative methods used to identify the social processes that underlie healthcare.

**Interaction:** Encounter between physician and patient helps in bringing together conflicting views of health and illness.

**Bias:** Bias is a systematic error. It leads to results which are consistently wrong in one/other direction. Bias leads to incorrect estimate of the effect of a risk factor/exposure.

**Confounding:** Describes the situation where an estimate of the association between an exposure and the disease is mixed up with the real effect of another exposure on the same disease, the two exposures being the same.

**Confidence interval:** A method of statistical inference that allows statement to be made about the publication using data from the sample.

**Odds ratio:** Ratio of exposure among cases to exposure among controls.

**Chance:** Chance/sampling error plays a role in most studies of humans, since it is rarely if ever possible to include an entire population in an investigation. We therefore attempt to infer information about the population on the basis of information obtained from representative samples drawn from the population.

**Naturalism:** Qualitative methods seek to understand health and health-related behavior in its every day or ‘natural’ context.

### Why is it so difficult to get good evidence on a subject?

- Inadequate steps to control bias.[6]
- Insufficient number of participants studied.
- Ignoring questions and outcomes of interest to patients.
- Lack of rigorous scientific data to support clinical practices.

Evidence-based approach in periodontal therapy will be dealt under the following topics:

- EBA and mechanical nonsurgical pocket therapy
- Effect of smoking on NST
- EBA in periodontal regeneration
- EBP and open flap debridement
- EBA and mucogingival surgery
- EBA and dental implants

### Evidence-based approach and mechanical nonsurgical pocket therapy

A total of nine reviews were searched for the best evidence.[7]

- Nonsurgical pocket therapy (NST) was found to have a positive effect with the exception of pockets <3 mm.
- Patient, environmental, and operator factors affect therapy delivery.
- No difference was found between the effect of hand and machine-driven instruments.
- Machine-driven instruments were faster than hand-driven instruments.

### Conclusions from 1996 world workshop on periodontics

#### Chemical plaque control[8]

- The various antiplaque and/or antigingivitis agents do not offer a substantial benefit for the treatment of periodontitis.
- They may however contribute to the control of gingival inflammation that exists with periodontitis.
- Supragingival irrigation may be used as an adjunct to toothbrushing and has been shown to aid in the reduction of gingival inflammation.
- Even when subgingival irrigation is used, the evidence shows that there are no clear substantial long-term benefits for the treatment of periodontitis.

#### Antibiotic therapy and periodontics[8]

The risk–benefit ratio indicates that systemic antibiotics should not be used for the treatment of gingivitis and common forms of adult periodontitis. But evidence suggests that systemic antibiotics may be useful in aggressive forms of periodontitis.

#### Local delivery of antimicrobial agents[8]

- There was modest gain in clinical attachment level and decrease in probing depth and gingival bleeding.
- A few side effects were demonstrated namely, transient discomfort, erythema, recession, allergy, and rarely, candida infection.

### Implications for future research

1. Effect of NST in different population groups is to be estimated.
2. Operator aspects should be included in therapy effectiveness.
3. Patient-oriented research to be conducted.
4. Efficiency studies performed.
5. Use of NST in maintenance treatment to be investigated.
6. Researchers should provide details of study design, conduct, and analysis.
7. Future studies should be designed to be incorporated in future systematic reviews.

**Table 1:** Levels of evidence

Level	Type of evidence[4]
1a	Systematic review of RCT
1b	Individual RCT
2a	Systematic review with homogeneity of cohort studies
2b	Individual cohort study
2c	Ecological studies
3a	Systematic reviews with homogeneity of case-control studies
3b	Individual case–control study
4	Case-series
5	Expert opinion without explicit critical appraisal

It was concluded that though adjunctive therapies continue to be explored, mechanical debridement is still the single best option available. It remains the foundation treatment for many adjunctive antimicrobial treatment investigations.

### Effect of smoking on nonsurgical therapy

Systematic review of the effect of smoking on NST was conducted by Labriola *et al*.[9] Search strategy included Medline, Embase and Central. Study design was controlled clinical trial. The outcomes were:

- There was reduced pocket depth reduction in smokers, compared with nonsmokers.
- There was no significant difference in the change of Clinical Attachment Level (CAL) between smokers and nonsmokers.
- The reason could be that the increased vasoconstriction in peripheral blood vessels of smokers leads to decrease in bleeding and edema. Also, smokers would have less potential for resolution of inflammation and edema within the marginal tissues and therefore less potential for gingival recession.

### Evidence-based approach in periodontal regeneration

#### Guided tissue regeneration

The study population included chronic periodontitis patients in subjects 21 years or older. The outcomes assessed were:

#### Short-term clinical outcomes

It included soft tissue changes such as increased CAL and decreased PPD.

#### Long-term clinical outcomes

It included disease recurrence and tooth loss.

#### Patient-centered outcomes

It included various factors such as ease of maintenance, change in esthetics, p/o complications, cost/benefit ratio, and patient well-being.

The meta-analysis done by Needleman *et al*[10] and Murphy *et al*,[11] revealed that:

1. When compared with OFD, guided tissue regeneration (GTR) showed increase in CAL, decrease in PPD, and defect fill.
2. When GTR with bone substitutes was compared with GTR alone, the results were similar.
3. No evidence was found for difference in use of ePTFE versus bioabsorbable membranes.
4. Long-term clinical outcomes/patient-centered outcomes could not be determined due to lack of available data. Heterogeneity was large and bias could not be eliminated.

### Grafting procedures

Meta-analysis was done by Reynolds *et al*[12] and Trombelli *et al*.[13]

The therapeutic end points used were

- Short-term changes [12 months after intervention]
- Long-term changes [13 months or more]
- Patient-oriented changes

### Short-term changes

#### Autogenous bone

Trombelli *et al*,[13] in his review demonstrated greater CAL gain in autogenous graft group than the control group, but the result was not statistically significant. Reynolds *et al*,[12] showed a statistically significant gain in CAL.

#### Bone allograft

Use of bone allograft showed gain in CAL, PPD reduction and increased defect fill.

#### Dentin allograft

Use of dentin allograft showed a gain in CAL of 2.8 mm in grafted patients as compared with 2 mm CAL gain in controls.

#### Coralline calcium carbonate

Use of the graft showed a gain in CAL and bone fill. But there was no improvement in pocket depth reduction.

#### Bioactive glass

There was improvement of bony lesion when compared with open flap debridement [OFD]. Mean difference in CAL between the two was 1.04mm. Change in bone fill noted was greater for bioactive glass, but the change was not statistically significant. Heterogeneity was present due to a study conducted by Org *et al*,[14] which demonstrated a more favorable change following an OFD procedure.

### Porous/nonporous hydroxyapatite Polymethyl methacrylate (PMMA) and polyhydroxyethylmethacrylate (PHEMA)

#### Polylactic acid granules

All these graft materials, showed gain in CAL and decrease in probing pocket depth.

### Long-term outcomes

Fleming *et al*,[15] did a 6–36 months follow-up study and found that there was 0.12 mm gain in clinical attachment level gain in test group and 0.43mm decrease in clinical attachment level in control group. Galgut *et al*,[16] assessed and compared clinical attachment level at 12 months and 48 months. The results showed a 0.27mm decrease in clinical attachment level in grafted group and 0.14mm gain in clinical attachment level gain in open flap debridement group. Yukna *et al*.[17] followed-up hydroxyapatite grafted patients for a period of five years. The results showed that two-thirds of the patients showed a gain in clinical attachment level in the grafted group and one-third of open flap debridement showed a decrease in clinical attachment level.

### Patient-centered outcome

In most of the studies reviewed, there were no systemic or local adverse effects.

The adverse effects noted in some of the studies were

- Pebbled surface texture of grafted site
- Transient slight gingival inflammation
- Delayed soft tissue healing
- Exfoliation/shedding of graft material

### Heterogeneity of grafting material

- Heterogeneity observed in the various studies might be due to other factors.
- Studies, which support bone fill in open flap debridement procedure.
- No report of outcomes in smokers and nonsmokers.
- Plaque did not play a significant role in all these studies, since the patients were under strict oral hygiene during the procedure.
- Variability in outcomes and variability in results was noticed.
- Technique-sensitive grafting procedures.

So, it could be concluded that

- All grafts produce CAL gain, decrease in PPD, and bone fill, except polylactic acid.
- There was considerable heterogeneity in the studies.
- The studies could not tell treatment-related adverse effects and cost–benefit ratio.

### Emdogain

The main advantage of emdogain is formation of acellular cementum.

### Outcomes measured

- Primary outcome
- Secondary outcome
- Long-term benefits
- Patient-centered outcomes

Esposito *et al*,[18] conducted the systematic reviews and found that when emdogain was compared with open flap debridement, the results favored emdogain. When emdogain was compared with GTR, GTR was better in relation to decrease in probing pocket depth.

### Heterogeneity of emdogain

- Heterogeneity observed was mainly due to
- Antibiotics given
- Surgical technique used in control group
- Funding by manufacturer
- Risk of bias
- Baseline depth of intrabony defects

### Drawbacks of emdogain

- Gel-like consistency which limits the space making
- If primary closure is not assured, displacement of material takes place
- Adequate preservation of interdental soft tissue to limit collapse of flap into bony defect

### Concluding remarks

- Emdogain showed an improvement in CAL gain and PPD reduction.
- General conclusions about clinical relevance are limited.
- There is no difference between emdogain and GTR except for slightly increased reduction in pocket depth due to increased recession in GTR treated sites.
- Long-term effects are unknown.

### Evidence-based approach and open flap debridement

Sytematic reviews were conducted by Heitz Mayfield *et al*[19] and Antczak *et al*.[20]

### Clinical implications of the whole review regarding open flap debridement

If pocket depth reduction is the main aim, surgical treatment is the treatment of choice.

If increase in clinical attachment level gain is the main aim, nonsurgical therapy is of more benefit for shallow and moderate pockets and surgical therapy is the treatment of choice for deep pockets. Predictability of treatment outcome at sites with furcation involvement or angular defect is unclear.

### Evidence on mucogingival therapy

Carlo Clauser[21] in his meta-analysis found that:

- All the surgical procedures allow complete root coverage.
- Connective tissue grafting achieves complete root coverage more frequently than does GTR.
- The probability of complete root coverage is high if the initial recession is shallow, irrespective of the surgical procedure employed.
- The probability of achieving complete root coverage decreases dramatically as the initial recession depth increases.

Critical review by Pagliaro[22] on surgical root coverage led to the following conclusions:

- The overall clinical outcome of different techniques appears to be satisfactory, but the great variability among different studies creates difficulties in deciding which procedure is best suited for each clinical situation.
- The data are quite heterogeneous.
- The data are seldom eligible for further comparative analysis even after some missing data are computed.
- The editors of periodontal journals could promote decisive measures for establishing clear mandatory standards for presenting data in research articles.

### Dental implants

- Most evidence is available for titanium implants, but some evidence exists to support the use of hydroxyapatite and titanium-plasma sprayed implant surfaces.[23]
- There is also evidence to support the use of both two-stage systems which require a second surgery to expose the implant, and one-stage implant systems.
- Clinicians should exercise caution when treating patients who smoke and those with untreated periodontal diseases, poor oral hygiene, uncontrolled systemic disease and a history of radiation therapy in the region or active skeletal growth.

### Need for studies reporting individual patient data[21]

Individual patient data is considered the gold standard for the following reasons:

- Only IPD can provide the information needed to investigate the role of various factors in different clinical situations.
- If data are only available on a trial level and not for individual sites, it is impossible to individually relate the baseline recession depth of a site to the treatment results of that specific site.

### What is the significance of individual patient data?[21]

The clinical trial usually answers yes or no, but the rest of the information remains unused. The lost information would be very valuable in exploring data in order to raise few sensible questions and to design new trials. Therefore at least the following issues are relevant:

- The possibility of exploring data from different viewpoints.
- The possibility of analyzing the same data in different ways.
- The possibility of replicating the study to reduce the margin of doubt that cannot be eliminated.
- The possibility of an in-depth check of the reliability of the data collection and analysis.
- The possibility of sizing new experiments in an economically sound way by saving or designing expensive pilot studies more rationally.
- The possibility of computing the confidence intervals of some statistics those are of interest to the reader.

### New pathway for scientific articles[21]

- Submittal of a ‘conventional’ paper with summarized data.
- Provisional acceptance: The author could even choose between submitting the original set of data prior to publication or accept the challenge of confronting the editor's criticism of the published paper.
- The ‘conventional’ paper is published in the journal. The original data and other elaborations by the authors are published on the journal's internet site.
- A forum to promote discussion of the article via e-mail could be created and new ideas could certainly be a valuable by-product.

### Future

The aim of any future study should be to provide a maximum of useful information for both the practitioner and the researcher while reducing the cost of improving our knowledge. Any future article should report at least a minimum set of relevant data, as outlined in the companion paper. Moreover, individual patient data should be made available for readers and reviewers.
